# Daily Encounters of Mental Illness Stigma and Individual Strategies to Reduce Stigma – Perspectives of People With Mental Illness

**DOI:** 10.3389/fpsyg.2020.590844

**Published:** 2020-10-30

**Authors:** Wei Jie Ong, Shazana Shahwan, Chong Min Janrius Goh, Gregory Tee Hng Tan, Siow Ann Chong, Mythily Subramaniam

**Affiliations:** Research Division, Institute of Mental Health, Singapore, Singapore

**Keywords:** mental illness stigma, individual strategies, reducing stigma, public stigma, structural stigma, Singapore, qualitative, patients perspective

## Abstract

**Introduction:**

A qualitative evaluation of mental illness stigma experienced by people with mental illness (PMI) is currently lacking in Singapore. This study aims to employ qualitative methods to identify the common encounters of mental illness stigma experienced by PMI in Singapore and uncover their individual strategies and efforts to reduce mental illness stigma.

**Methods:**

This study is part of a larger research project that explores the concept of mental illness stigma among different stakeholders in Singapore. Focus group discussions (FGDs) were conducted with 42 PMI to collect qualitative data on their experience with mental illness stigma, including encounters of stigma and individual strategies to reduce stigma. The inductive thematic analysis method was employed to analyze the data.

**Results:**

The eight emergent themes associated with encountering stigma in PMI’s everyday life were categorized into two over-arching themes, public stigma (i.e., negative beliefs and attitudes, subjected to contemptuous treatment, social exclusion, over-scrutinizing, and receiving excessive care and concern) and structural stigma (i.e., the requirement to declare psychiatric conditions during job interviews, excluded from consideration after the declaration, and requirement of medical endorsements for employment). Four themes regarding PMI’s individual strategies to reduce stigma were also identified (i.e., non-disclosure of condition, standing up for themselves, individual efforts in raising awareness, improving themselves, and living life as per normal).

**Limitations:**

Participants may be influenced by social desirability bias due to the presence of other participants in an FGD setting. Also, those who agreed to participate in the study may possess strong views or beliefs about mental illness stigma and may therefore be inherently different from those who refused to participate.

**Conclusion:**

Our findings on instances of public and structural stigma encountered by PMI in Singapore can guide policymakers with the development of future policies and strategies to reduce mental illness stigma in the Singapore society. Furthermore, our study also identified individual strategies that PMI employed to reduce mental illness stigma. However, the effectiveness of these strategies was unclear and little is known of their effect on PMI themselves. Hence, there is a need for future studies to examine these strategies.

## Introduction

The stigma of mental illness is ubiquitous and found consistently across different cultures (Angermeyer and Dietrich, 2006; Thornicroft et al., 2009). Mental illness stigma could lead to various ramifications such as, but not limited to, negative impacts on help-seeking, treatment adherence, self-esteem, and quality of life (Alonso et al., 2009; Livingston and Boyd, 2010; Henderson et al., 2013). Public, structural, and self-stigma are some of the different constructs of stigma described in the current literature (Corrigan and Bink, 2005; Rüsch et al., 2005). Public stigma is defined as the endorsement of stereotypes, prejudices, and acts of discrimination toward people from a stigmatized group (Corrigan et al., 2004; Rüsch et al., 2005). Common stereotypes faced by people with mental illness (PMI) include the beliefs that they are dangerous, unpredictable, and incompetent (Angermeyer and Dietrich, 2006; Parcesepe and Cabassa, 2013). These stereotypes can lead to negative attitudes, such as fear and uncertainty (Corrigan and Bink, 2005; Angermeyer and Dietrich, 2006; Parcesepe and Cabassa, 2013). Furthermore, PMI are also subjected to discriminatory behaviors like social exclusion or not been taken seriously by others (Parcesepe and Cabassa, 2013; Mestdagh and Hansen, 2014). Structural stigma comes in the form of institutional policies that intentionally or unintentionally restrict the opportunities of people from the stigmatized group (Corrigan et al., 2004; Rüsch et al., 2005). Examples of structural stigma include the requirement to disclose the history of mental illness during school and job applications, reducing one’s privacy, and discrimination over job opportunities due to one’s mental illness (Suto, 2012; Pugh et al., 2015). PMI may also internalize the public and structural stigma experienced in their daily life leading to self-stigmatization (Corrigan and Watson, 2002).

Existing literature has highlighted the importance of cultural influences on the expression of mental illness stigma and consistently identified cultural differences in terms of stigmatizing beliefs and attitudes toward PMI across different countries (Angermeyer and Dietrich, 2006; Yang et al., 2007; Abdullah and Brown, 2011; Cheon and Chiao, 2012). For example, Asian families may develop a sense of shame toward family members with mental illness resulting from their collectivist nature, and people of African descent are likely to perceive PMI who are unable to take on different roles within a family as irresponsible or unreliable due to the significance of role flexibility in their culture (Abdullah and Brown, 2011). More importantly, the literature on mental health literacy and anti-stigma interventions has highlighted the need for culturally and contextually developed interventions (Dalky, 2012; Kutcher et al., 2016). Hence, it is vital to obtain contextual information about mental illness stigma encountered by PMI in a specific culture in order to inform future interventions. This can be better achieved via qualitative methods instead of quantitative methods that use pre-defined questions and hypothetical situations.

Singapore is a multi-ethnic country with a total population of four million residents that comprise 74.4% Chinese, 13.4% Malays, 9.0% Indians, and 3.2% of other ethnicities (Singapore Department of Statistics, 2019). Although multiple quantitative studies have been conducted locally and have consistently found considerable mental illness stigma toward PMI across Singapore, an in-depth qualitative understanding of mental illness stigma encountered by PMI in Singapore is currently lacking (Lai et al., 2001; Picco et al., 2017; Subramaniam et al., 2017). Therefore, for a country with a unique blend of traditional beliefs and cultures rooted in the local community, it is necessary to explore and understand the lived experience of mental illness stigma by PMI residing in Singapore.

Thus, this study aims to conduct focus group discussions (FGDs) to identify the everyday encounters of mental illness stigma (i.e., public and structural stigma) experienced by PMI in Singapore. Also, as little is known about how PMI in Singapore respond to mental illness stigma, this study seeks to explore PMI’s individual strategies and efforts to reduce mental illness stigma.

## Materials and Methods

This study is part of a larger research project that explores the concept of mental illness stigma among different stakeholders (i.e., the general public, PMI, caregivers of PMI, healthcare professionals, and policymakers/influencers) in Singapore. This study utilized the qualitative data collected from PMI. The study was approved by the National Healthcare Group Domain Specific Review Board.

### Sample

A total of 42 PMI, aged 21 years and above were recruited between March 2018 to May 2018 through convenience and snowball sampling. As Singapore is a multi-ethnic country with English being the common language of use across different ethnicities, we recruited participants who were conversant and literate in English. Furthermore, we acknowledged that the encounter and degree of stigma experienced by PMI might vary depending on their diagnosis (Subramaniam et al., 2017). Hence, we recruited patients with two specific diagnoses only, i.e., psychotic disorders and mood disorders, to ensure a homogenous account of the encounters of stigma amongst PMI. All participants provided their written, informed consent before the commencement of data collection.

### Data Collection

Data collection was done via FGDs. Participants were grouped according to their diagnosis (i.e., psychotic disorder group and mood disorder group) to ensure homogeneity in the FGD groups. This can help participants feel more comfortable while expressing their opinions. A total of six FGDs were conducted (three psychotic disorder groups and three mood disorder groups). Each FGD comprised 5–8 participants and lasted between 1.5 and 2 h. The FGDs were conducted in a meeting room within a community center to ensure the neutrality of the venue. At the start of the session, background information (i.e., age, gender, education level, ethnicity, religion, diagnosis, and age of diagnosis) was collected from the participants with a socio-demographic form. Each FGD was conducted by a facilitator with a note taker present. The facilitators were trained and experienced in qualitative research methodologies. The topic guide that was developed by the study team was used for all the FGDs. The topic guide consisted of open-ended questions that explored various areas of mental illness stigma such as encounters of stigma, reasons for stigma, individual strategies to reduce stigma, knowledge and comments on existing intervention for mental illness stigma, and suggestions for future interventions. The FGDs were audiotaped and transcribed verbatim for analysis.

### Analysis

The data were analyzed with an inductive thematic analysis method (Braun and Clarke, 2006). Transcripts were first distributed amongst five study team members (WJ, SS, GT, JG, and MS) for familiarization with the collected data. Subsequently, each study team member independently identified preliminary codes from their respective transcripts. The study team members then came together and generated themes through an iterative process of sorting the collated preliminary codes into potential themes, assessing the congruency of code within the themes, and ensuring there was no overlap between the themes. A codebook was developed with the derived themes to guide the coding process. To ensure consistency of coding among the study team members, the same transcript was coded to establish inter-rater reliability. The study team continued to discuss, refine the codebook and repeated the coding with another transcript until a satisfactory inter-rater reliability score was achieved (Cohen’s Kappa score >0.75). After coding three transcripts, Cohen’s kappa was established at 0.77. Transcripts were then distributed among the study team members for coding. Data analysis was completed with Nvivo Version 11.0.

## Results

Participants were between 21 and 58 years old. The majority of them were female (57.1%), and of Chinese ethnicity (64.3%). Additionally, the number of participants diagnosed with a mood or psychotic disorder were 18 and 24, respectively. Socio-demographic information is displayed in Table 1.

**TABLE 1:** Socio-demographic characteristic of the participants.

Variable	Range	Mean
Age (in years)	21–58	33.4
	***N***	**Percentage**
**Sex**		
Male	18	42.9
Female	24	57.1
**Ethnicity**		
Chinese	27	64.3
Malay	10	23.8
Indian	4	9.5
Others	1	2.4
**Education level**		
Secondary school/O/N level/completed secondary education	7	16.7
A level/completed pre-U or junior college	4	9.5
Vocational institution/ITE Nitec Cert	4	9.5
Polytechnic diploma	12	28.6
Other diploma	6	14.3
University degree	7	16.7
Post-graduate degree (e.g., masters/Ph.D.)	1	2.4
**Diagnosis group**		
Mood disorder	18	42.9
Psychotic disorder	24	57.1

*1 missing response for Education level (2.4%).*

### Daily Encounters of Mental Illness Stigma

A total of eight broad themes associated with encountering stigma in PMI’s everyday life emerged from the analysis (refer to Table 2 for the frequency of reoccurrence of the themes amongst the FGDs). The themes were categorized into two over-arching themes, (1) public stigma and (2) structural stigma.

**TABLE 2:** Frequency of reoccurrence of themes amongst the FGDs.

Identified themes	Frequency of reoccurrence amongst FGDs
**Public stigma**	
Negative beliefs and attitudes	6
Subjected to contemptuous treatment	5
Social exclusion	4
Over-scrutinizing	4
Receiving excessive care and concern	3
**Structural stigma**	
The requirement to declare psychiatric condition during job interview	2
Excluded from consideration after declaration	3
Requirement of medical endorsements for employment	2
**Individual strategies to reduce stigma**	
Non-disclosure of condition	3
Standing up for themselves	3
Individual efforts in raising awareness	6
Improve themselves and live life as per normal	4

#### Public Stigma

##### Negative beliefs and attitudes

Participants indicated that they were perceived negatively by others in their daily lives. Three sub-themes relating to negative beliefs and attitudes were identified: (1) dangerous, unpredictable, and simply crazy, (2) inferior and incapable, and (3) having a character flaw instead of a medical disorder.

###### Dangerous, unpredictable, and simply crazy

It was believed by the participants that the general public held a firm belief that PMI possessed a great tendency toward violence. They were also perceived as volatile, mentally unstable, and having a high propensity to act in sudden and unexpected ways. Furthermore, it was not uncommon for PMI to be seen crudely as being simply crazy, regardless of their diagnosis. Consequently, as believed by the participants, these negative perceptions had brewed fear and wariness among the general public toward PMI.

“*Mental illness equals mental instability and mental instability means everything also unstable, means you are even more prone to attack.” (Mood Disorder Group 2)*

“They probably feel fearful toward you cause they don’t know what to expect.” (Psychotic Disorder Group 1)

“Even worse, sometimes it’s that people generally think crazy is crazy. There’s no such thing as bipolar, no such thing as schizophrenia, depression. They just label it as under one which is crazy.” (Mood Disorder Group 3)

###### Inferior and incapable

As expressed by the participants, it is common for PMI to be looked down upon by others across various settings. People felt that PMI were inferior, incapable, and could not be trusted with responsibilities. Some participants articulated that they were often seen by employers as being less competent at work as compared to other colleagues. Moreover, PMI were also viewed as having poor prospects in life. Parents, too, were reported to develop a sense of shame toward their children with mental health problems for such reasons.

“I think people may think that those with mental illnesses are not capable of doing things as what the others can do.” (Psychotic Disorder Group 1)

“I think it is more like negative meaning like the person couldn’t achieve much in life, couldn’t live a normal life, like other normal people, don’t have a family and so forth.” (Psychotic Disorder Group 1)

“I think people are ashamed to admit that or to address, even as a parent for you to say to another parent that my child is mentally ill. That is going to be equals to he’s not going to fare well in his exams.” (Mood Disorder Group 1)

###### Having a character flaw instead of a medical problem

Participants complained that PMI were perceived as possessing character flaws by the public such as being lazy or weak. PMI were frequently perceived as being lazy by teachers and their family members when accompanying symptoms of their illness, such as avolition affected their ability to complete their school work or look for a job, respectively. Furthermore, it was believed that PMI were weak, and hence they were not capable of handling life stresses which resulted in their mental illness.

“I think people have this misconception that most of the time mental illness and psychological disorders are a character problem.” (Psychotic Disorder Group 3)

“The teachers don’t really know or understand what you’re going through. So, they tend to just look at me as like oh lazy, never do homework or they just have this very bad idea of me like they don’t really know what’s going on so they kind of judge me as the bad student.” (Psychotic Disorder Group 3)

“You know, you’re just weak you know. Just toughen up”, if we all can rise through difficult situations… you know especially my mom, she went through divorce when she was 35 with 4 kids. So she said that to me if I could go through something so tragic like that, why for you, you’re not even married and you know you’re just going through breakups and stuff like that so why are you behaving this way. (Mood Disorder Group 1)

##### Subjected to contemptuous treatment

As highlighted by some participants, it is not uncommon for PMI to be at the receiving end of a range of contemptuous treatment by their family members, relatives, and healthcare professionals. Some participants complained about family members treating them with disrespect, such as using stigmatizing language and making threats against them. Furthermore, some of the participants felt that they were regarded as the object of ridicule by their relatives. One of the participants described that her relatives tried to intrude in her personal matters to satisfy their own curiosity and for their amusement. Participants also pointed out instances where healthcare professionals behaved rather unkindly toward them. In addition, their opinions were often not taken seriously by healthcare professionals.

“So he(brother) will use the mental illness to agitate me even more and say that I can go and stay in the hospital or all that when I am actually perfectly alright.” – Psychotic Disorder Group 1

“They(relatives) are not like sympathetic like they want to help or what you know. They just want to satisfy their curiosity, that’s all. They are… even like visiting, she(mother) feels like my relative will see this place like a circus” (Psychotic Disorder Group 1)

“There was this nurse there, that like was openly being rude about other patients in the wards… toward my friend. Calling them lunatics.” (Psychotic Disorder Group 3)

##### Social exclusion

Participants also experienced social exclusion in their daily life. Specifically, friends, acquaintances, and colleagues tried to avoid being in close proximity with PMI and were reluctant to form any social relationship with them upon learning that they had a mental illness.

“I think they doesn’t want a friend in their list that is labeled as mental illness.” (Mood Disorder Group 3)

“My colleague… I don’t like you, I don’t want to sit beside you kind of thing will really mentally torture me.” (Mood Disorder Group 3)

##### Over-scrutinizing

Participants stated that they were constantly scrutinized by those who knew about their condition. They provided instances where family members and friends overreacted and associated their day-to-day behaviors as signs of relapse. Furthermore, it was also pointed out that some employers/supervisors were fixated on PMI’s condition and became overly critical of their work performance.

“Especially from your mom like she knows that I have bipolar and then it’s like she will always always always look like… there’s symptoms and see if I’m like… something wrong with me ah. Like sometimes I cannot sleep, sometimes I sleep too much, she will note down you know okay must be something wrong with him. Must be manic today, depressive today you know.” (Mood Disorder Group 3)

“The supervisor knows that I have mental issues and the way she treat me… she says “you are slow”, when in fact I am not. Her expectations are so much higher. It’s different, I can sense it.” (Psychotic Disorder Group 1)

##### Receiving excessive care and concern

It was highlighted by participants that some people who knew about their condition expressed excessive care and concern. Although these people had good intentions, participants saw it as being “over-compensating” and instead felt that such acts were stigmatizing. As described by participants, this behavior can be demeaning, discomforting, and sometimes intrusive.

“Because the overcompensation is… it makes me feel suffocated ah you know, people constantly checking are you okay? Are you okay? Are you okay? Then I like feel like what, what is this? You know I’m not like retarded or anything like that.” (Mood Disorder Group 3)

“I do feel that they’re overcompensating slightly as he said in that sense because they’re offering stuff like okay if you let’s say you have bipolar disorder correct, err if you are having a depressive phase, they you know may extend your assignment deadline.” (Mood Disorder Group 3)

“We don’t know each other very well. But they (colleagues) will be too concerned about me. They will ask about how I’m doing, do you feel better, but actually they don’t know about me at all. I don’t want to share with my, the things with them. So I’ll feel a bit weird. They will try to feel very close to me but actually not so close.” (Psychotic Disorder Group 3)

#### Structural Stigma

Participants reported encountering structural stigma primarily within the employment setting.

##### The requirement to declare psychiatric condition during a job interview

As mentioned by the participants, some job interviews required applicants to declare any history of psychiatric conditions via a declaration form. Participants perceived this practice as stigmatizing. They reasoned that having a history of psychiatric conditions does not determine one’s ability to work; hence, it was not necessary for employers to collect this piece of information.

“Sometime I don’t understand also why when you have an application form you must declare mental illness. Why is it an issue about… so if you have, what does it say? You think the person won’t perform on the job, why not? I mean you still go through interview process.” (Psychotic Disorder Group 1)

##### Excluded from consideration after the declaration

Participants also felt that once they declared their psychiatric illness during job interviews, they would procedurally be removed from consideration. Specifically, interviewers lose interest in them and abruptly end the interview. As believed by the participants, employers and human resources departments operate in the interest of the business and hiring a PMI was perceived as a risk to their business.

“During job interview, once I declare my condition they cut short the interview and say oh we’ll let you know if we would like to proceed on with your application.” (Psychotic Disorder Group 3)

“So HR unfortunately again, if the businesses are run purely on business profit, then HR will always raise that question (hiring PMI).” (Mood Disorder Group 2)

##### Requirement of medical endorsements for employment

Some participants shared that they were required to provide endorsements such as a letter from physicians to certify that they were fit for work in order to be employed or continue their employment.

“I was dealing with children so, she actually needs letter to prove that I am actually fit for work. If not she will think I’m violent or whatever it is due to my illness. So what we need is the proof.” (Psychotic Disorder Group 2)

“Since 2010 they ask me to see psychiatrist so every year they ask for my medical report.” (Mood Disorder Group 3)

### Individual Strategies to Reduce Stigma

The analysis also found four themes regarding the individual strategies that PMI employed to reduce mental illness stigma (refer to Table 2 for the frequency of reoccurrence of the themes amongst the FGDs).

#### Non-disclosure of Condition

To avoid being subjected to mental illness stigma, many participants chose not to disclose their condition. Participants felt that there was no need to tell others about their mental illness as many would not empathize, which could result in unnecessary problems and disclosure was unlikely to yield any benefits. A participant also commented that it was easier to get a job without disclosing their mental illness.

“I don’t talk about it, I don’t explain it. Explaining it people won’t believe you what, right or not, so don’t talk about it and then you don’t need to explain anything. So, there won’t be any stigma.” (Psychotic Disorder Group 3)

“From then onwards I didn’t declare and it’s easier to get the job.” (Mood Disorder Group 3)

#### Standing up for Themselves

Some participants commented that they stood up for themselves when they encountered mental illness stigma by confronting the perpetrator’s stigmatizing behavior/attitudes.

“because I’m a very straight forward person. I just told them that, your close-mindedness has got to go. Yeah, it’s… I’ve had enough of whatever you guys have had to say. I’m sharing with you how I feel, then if you’re not going to take it then I’m not going to talk.” (Mood Disorder Group 1)

#### Individual Efforts in Raising Awareness

To reduce mental illness stigma, many participants had made an effort to educate others, especially their families and friends, on mental illness, their experiences with the condition, and how to better communicate with them. Furthermore, a few of the participants also took on the role of more formal mental health advocates, such as appearing in a mental-health documentary.

“My thoughts on it I guess to help people understand what mental illness is, is the key. And as an individual I feel that I can do that as well. It’s not only you know the government or anybody else who should do it I feel it’s everybody’s responsibility.” (Mood Disorder Group 3)

#### Improve Themselves and Live Life as per Normal

Some participants sought to improve themselves and their condition and live their life as per normal to prove that they could also be a contributing member of society. As mentioned by some of the participants, they believed that they had to first change themselves before changing the opinion of others regarding PMI.

“Before we become the change in others we need to change ourselves first. So we need to get well, eat our medication and then get… resume our normal activities” (Psychotic Disorder Group 2)

“I just do the best, whatever I can. Work, try to act normal. Do my part in work, I just do my best in whatever I do to give others a good impression.” (Psychotic Disorder Group 3)

## Discussion

Our study has identified multiple themes of public and structural stigma. The themes categorized under public stigma were primarily associated with stereotypes, prejudices, and acts of discrimination. These themes were largely consistent with existing literature on mental illness stigma found across different countries.

Our study suggests that PMI are subjected to a range of stereotypes, and they are aware that the general public perceives them as dangerous, unpredictable, incapable or possessing a character flaw. Reviews of qualitative and quantitative literature have consistently identified being unpredictable, violent, and often in need of help as common stereotypes ascribed to PMI by the general public across countries (Angermeyer and Dietrich, 2006; Parcesepe and Cabassa, 2013; Mestdagh and Hansen, 2014). Sadler et al. (2012) also found that the general public in the United States rated PMI lowly on competency, comparable to poor people. Furthermore, the perception of PMI having a weak character is especially prevalent among Asian cultures due to their belief that it is a cause of mental illness (Abdullah and Brown, 2011).

PMI are also exposed to various prejudices from the general public, such as fear, wariness, and especially among family members, shame. Participants believed that these negative attitudes are consequences of the respective stereotypes held by the general public. Consistent with our findings, fear and uncertainty are well established in the literature as negative attitudes held by the general public against PMI internationally (Angermeyer and Dietrich, 2006). These are also frequently alluded to be a result of the stereotype that PMI are dangerous and unpredictable (Corrigan and Bink, 2005). Furthermore, studies have also highlighted that family members may perceive PMI as a source of shame for their family (Corrigan and Miller, 2004; González-Torres et al., 2007). Corrigan and Miller (2004) suggested that this prejudice stemmed from two types of stereotypes faced by the family members, the belief that family members are responsible for mismanaging their loved one’s condition and the transmission of mental illness from parents to the child. However, in our study, PMI shared that the shame experienced by family members was in relation to the beliefs that PMI are seen as incapable and their low prospects in life. This opinion might be specific to the Asian culture, especially among the Chinese, where “face” is a social construct rooted deeply in the Chinese culture. It can represent an individual’s or a family’s social status and standing in the community. As suggested by Yang and Kleinman (2008), due to the perception that people with schizophrenia are incompetent, having them in the family may lead to loss of “face.” Thus, family members may experience a sense of shame because having PMI in their family may have negative consequences on their family’s social status within the community.

Our study also identified daily instances of discriminatory behaviors encountered by PMI in various settings. Themes identified were, being subjected to contemptuous treatment, social exclusion, over-scrutinized, and receiving excessive care and concern. Our finding indicates that PMI are often faced with a range of contemptuous treatment by their family members and healthcare professionals. One example that came out strongly was the use of stigmatizing language toward PMI. A qualitative study conducted among healthcare professionals in Malaysia identified family and healthcare professionals as two of the most common perpetrators of mental illness stigma (Hanafiah and Van Bortel, 2015). Also, studies across cultures have consistently found that PMI are often subjected to name-calling and negative comments related to their condition in their daily life (Dickerson et al., 2002; Rose et al., 2011; Hanafiah and Van Bortel, 2015). On this note, participants had also complained that healthcare professionals could be rude and disrespectful to PMI. In addition, they felt that their opinions were often not taken seriously by healthcare professionals. In line with our findings, studies across cultures have also suggested that some healthcare professionals were guilty of talking down to PMI in a demeaning manner, involving PMI minimally with their own treatment experiences and frequently doubting PMI’s opinions (Thornicroft et al., 2007; Mestdagh and Hansen, 2014; Hanafiah and Van Bortel, 2015). Moreover, some of our participants also provided instances where they felt that they had been treated as a subject of ridicule by their relatives. Although few studies have explored PMI’s experience as a source of amusement and a target of mockery, it is documented in the literature that PMI are also often portrayed derisively in the media, apart from being dangerous (Stuart, 2006; Rose et al., 2011). This may have encouraged the perception that it is permissible for people to deride PMI.

Another theme that emerged from our study was social exclusion. Social exclusion has been extensively explored internationally as a form of discrimination toward PMI (Morgan et al., 2007; Parcesepe and Cabassa, 2013; Mestdagh and Hansen, 2014). Subramaniam et al. (2017) have also found that a sizeable percentage of the public in Singapore are unwilling to form social relationships with PMI. Our findings reiterate that the unwillingness to socialize with PMI is a universal obstacle faced by PMI in their everyday life across different cultures as posited by the literature.

Although over-scrutinizing was not identified widely in existing literature, it emerged as a theme associated with discriminatory behaviors amongst PMI in Singapore. Our participants highlighted that they were subjected to constant scrutiny by people who knew about their condition. Specifically, our participants provided examples such as family members being constantly on the lookout for telltale signs of relapse and work supervisors being fixated on PMI’s condition, which resulted in overcritical judgment with PMI’s work performance. These behaviors may be explained with the concept of confirmation bias (Nickerson, 1998). A study showed that caregivers of patients with psychosis were well aware of their care recipient’s risk of relapse (Chan et al., 2015). Moreover, as previously mentioned, PMI are often perceived as incompetent (Sadler et al., 2012). Hence, it is probable that people may actively seek out and interpret PMI’s behaviors as consistent with their respective beliefs such as proneness to relapse or incompetence.

Last but not least, PMI also complained that people expressed excessive care and concern toward them, which could affect their dignity and self-esteem. These findings resemble elements of both emotional over-involvement and infantilization, two types of maladaptive behaviors that care recipients may receive from their caregivers. Emotional over-involvement is often linked to a range of intrusive, overprotective, and self-sacrificing behaviors, whereas infantilization, is associated with the treatment of care recipients, especially older adults, as children such as using patronizing and overfamiliar languages (Singh et al., 2013; Marson and Powell, 2014). Consistently, studies across cultures have found that people with schizophrenia are exposed to infantilization, emotional over-involvement and forms of overprotection which may limit their privacy, personal growth and self-identity (González-Torres et al., 2007; Yang and Kleinman, 2008; Mestdagh and Hansen, 2014). It was also suggested that these behaviors again emanate from the perception that people with schizophrenia are incompetent (Yang and Kleinman, 2008). Our findings, suggest that such behaviors come not only from caregivers but also from others whom the PMI meet in various types of settings (i.e., work, school, and social setting). In general, the instances of public stigma experienced locally by PMI were generally congruous to findings across different cultures. However, as a country in Southeast Asia, the Asian culture seems to have a strong influence over the public stigma experienced by PMI in Singapore. Hence, local policymakers can reference existing policies and interventions implemented in Asian countries when formulating future policies and interventions.

Our participants reported encounters of structural stigma predominantly in the area of employment. First of all, they felt that they had been excluded from consideration once they disclosed their condition during a job interview. A systematic review examined the employment of people with disability (i.e., someone with a physical or mental impairment) from a human resource development perspective (Procknow and Rocco, 2016). An institutional barrier identified by the review was that employers may have economic concerns regarding the productivity of people with disabilities and deemed them as less attractive candidates (Procknow and Rocco, 2016). Therefore, organizations may be reluctant to hire PMI due to the perception that it is a financially risky decision. Secondly, our participants viewed the practice in which they were required to declare their psychiatric illness via a declaration form during job interviews as discriminatory and uncalled for. The relevant laws in various countries have disallowed this practice because of the possibility that it may expose people with disabilities to various unjustified employment barriers and dismissals (De Schutter, 2004). Only very recently, has this practice been disallowed in Singapore (Zhou, 2020). Participants also perceived the need to produce a “fit for work” medical endorsement from doctors for their employment as discriminatory. However, this practice may be necessary to safeguard the interest of PMI. Corrigan et al. (2004) cautioned that some institutional policies or procedures might restrict the opportunities of a certain group of people; however, they may still be justifiable and should not be deemed as discriminatory. A doctor’s assessment on patients’ fitness for work can help to identify patients who are not suitable for a particular type of employment which could prevent potential occupational hazards at work due to their illness (Coggon and Palmer, 2010). They could also provide employment advice (i.e., possible functioning difficulties faced by the patients and job modification recommendation) to both patients and employers (Coggon and Palmer, 2010). Overall, PMI in Singapore perceived strong structural stigma in the employment setting. Despite improvements made in recent years, more work needs to be done locally to reduce mental illness stigma amongst organizations (i.e., employer and co-workers). Regulation and policies need to be introduced to ensure equal opportunities for PMI and curtail the use of certain procedures required during employment which are stigmatizing.

Our study also identified individual strategies that PMI employed to reduce mental illness stigma in their daily life. Some PMI chose to conceal their condition to avoid being subjected to mental illness stigma. Concealment of one’s condition is commonly employed by PMI as a coping strategy against stigma (Holmes and River, 1998; Corrigan et al., 2013). As mentioned by Corrigan et al. (2013), there are both pros (e.g., avoid stigmatizing people and fewer concerns with others’ perception) and cons (e.g., less opportunity for social support and experience of guilt from concealing condition) of keeping one’s condition concealed. Hence, PMI who perceived the benefits greater than the costs subscribed to this strategy.

On the other hand, some PMI preferred to stand up for themselves against the perpetrators of discrimination. Discrimination is commonly regarded as an unjust behavior toward a certain group of people (Knight, 2013). Miller’s (2001) review suggested that victims of unjust treatment may seek justice through their own actions in order to challenge the threat, restore their self-esteem, and educate the offenders on their wrongdoings. Thus, when PMI encounter stigmatizing behaviors, some may be motivated and decide to retaliate against their perpetrators and demand appropriate treatment.

Furthermore, some PMI were also committed to raising mental health awareness amongst the general public, including their loved ones and friends, to reduce mental illness stigma. Lack of awareness and knowledge of mental illness is frequently identified as a cause of mental illness stigma (Shrivastava et al., 2012). A survey conducted amongst 300 psychiatric patients in Singapore to understand their perception of mental illness stigma and its contributing factors found that the majority of the patients endorsed a lack of knowledge of mental illnesses among the general public, hence indicating the need to increase public awareness on mental illnesses (Lai et al., 2001). This recognition by PMI may have motivated them to educate their family members and friends on mental illnesses and encouraged them to participate in public mental health advocacy in order to lessen mental illness stigma.

Lastly, to reduce mental illness stigma, PMI also sought to better themselves and continue with their life as per normal. While it is well-established that perceived mental illness stigma may result in self-stigmatization among PMI, research has postulated that some PMI remain empowered or feel indifferent toward stigma (Corrigan and Watson, 2002). It is suggested that this is influenced by how much PMI identify with their condition and their perception on the legitimacy of the stigma (Corrigan and Watson, 2002). Hence, PMI, who identified less with their condition and perceived the stigma as less legitimate, may be less attentive toward the stigma or more motivated to improve themselves in order to change others’ opinions of PMI.

Our study has some limitations. Firstly, our participants may be influenced by social desirability bias due to the presence of other participants in an FGD setting which may have resulted in them withholding their truthful opinions. To minimize this bias, participants were assured that there are no right or wrong answers to the questions and information shared will be kept strictly confidential. Secondly, participants who agreed to participate in our study may possess strong views or beliefs about mental illness stigma and therefore may be inherently different from those who refused to participate, thus affecting the representativeness of our sample.

Our findings have highlighted instances of public and structural stigma that PMI in Singapore encountered in various contexts of their daily life. This information can guide policymakers with the development of culturally appropriate policies and strategies to reduce mental illness stigma in the Singapore society and identification of potential audiences who may benefit the most from such interventions. Furthermore, our study also identified individual strategies which PMI employed to reduce mental illness stigma. Although studies have established some effectiveness of large scale interventions such as education and contact-based programs in reducing mental illness stigma (Rüsch et al., 2005; Dalky, 2012), it is unclear whether individual versions of these approaches by PMI will achieve the same results. Moreover, it is also not known whether these individual strategies are beneficial and adaptive to PMI themselves. Hence, there is a need for future studies to examine the effectiveness of these individual strategies and their possible impact on PMI.

## Data Availability Statement

The datasets presented in this article are not readily available because participants did not consent to this data being shared publicly. Requests to access the datasets should be directed to MS, mythily@imh.com.sg.

## Ethics Statement

The study was reviewed and approved by the National Healthcare Group Domain Specific Review Board, Singapore. The patients/participants provided their written informed consent to participate in this study.

## Author Contributions

WO wrote the first draft of the manuscript. MS, SC, and SS designed the study protocol. MS, SS, WO, CG, and GT were involved in the development of the topic guide, collection of qualitative data through focus group discussions, and analysis of the data with inductive thematic analysis method. All authors gave their intellectual input to the manuscript and have read and approved the final draft of the manuscript.

## Conflict of Interest

The authors declare that the research was conducted in the absence of any commercial or financial relationships that could be construed as a potential conflict of interest.
